# Data set for transcriptome analysis of *Apocynum venetum* L.

**DOI:** 10.1016/j.dib.2018.08.207

**Published:** 2018-09-05

**Authors:** Ping Chen, Gang Gao, Chunming Yu, Jikang Chen, Kunmei Chen, Aiguo Zhu

**Affiliations:** Institute of Bast Fiber Crops, Chinese Academy of Agricultural Sciences, Changsha 410205, China

## Abstract

In this paper, we present the transcriptome profiles of the *A. venetum* L. by RNA-Seq approach. A total of 6.57 Gb raw data were obtained, and 52,983 unigenes with an average length of 1009 bp and N50 of 1632 bp were annotated with the 7 databases. The unigenes annotated to KEGG database were divided into 21 categories from 6 main groups. Among these, 4952 (22.21%) unigenes were clustered to “Global and overview maps”, and 1834 (8.23%) unigenes were clustered to “Carbohydrate metabolism”. In addition, 6340 unigenes containing 7579 SSRs were identified and the mononucleotide, dinucleotide, trinucleotide motifs were the most common motif type (95.59%), accounting for 39.62%, 36.02%, and 19.95%, respectively.

## **Specifications table**

**Table:** 

Subject area	Biology
More specific subject area	Plant biology; Bioinformatics
Type of data	Table, text file, graph, figure
How data was acquired	RNA sequencing, Illumina HiSeq. 2000 and BGISEQ. 500 platform
Data format	Raw
Experimental factors	The leaves of *A. venetum* were collected for RNA sequencing
Experimental features	The sterilized seeds of *Apocynum venetum* L. were allowed to germinate and grow for 30 days in half-strength MS agar medium inside a growth chamber with a 14 h light/10 h dark cycle, air temperature of 25 °C, photon flux density (PFD) of 280 mol m^−2^ s^−1^. The leaves of *A. venetum* were collected, immediately frozen in liquid nitrogen, and stored at -80 °C until use. In order to increase the transcriptome coverage, a mixture of samples from these chambers were pooled for RNA sequencing.
Data source location	The seeds of *A. venetum* were collected in Xinjiang Province, China
Data accessibility	Data are with this article and available at https://www.ncbi.nlm.nih.gov/sra/SRP151546.
Related research article [1]	Xie W, Zhang X, Wang T, Hu J. Botany, traditional uses, phytochemistry and pharmacology of *Apocynum venetum* L. (Luobuma): a review. *J. Ethnopharmacol.*, 2012;141(1): 1–8.

## Value of the data

• *Apocynum venetum* (luobuma) is a common fiber and medicinal plant widely distributed in the salt marish, desert margins, alluvia flats and riversides [2], [3], which makes it an invaluable model for bast fiber development and plant stress resistance research.
• The genetic information and gene sequences about the *A. venetum* in public databases are scanty.
• The large dataset of transcripts and unigenes can be useful as it provides abundant genetic information for identifying of *A. venetum* genes.
• The unigenes obtained provide a good resource for SSRs application in evolutionary genetic from *A. venetum*.

## 1 Data

**Fig. 1:**
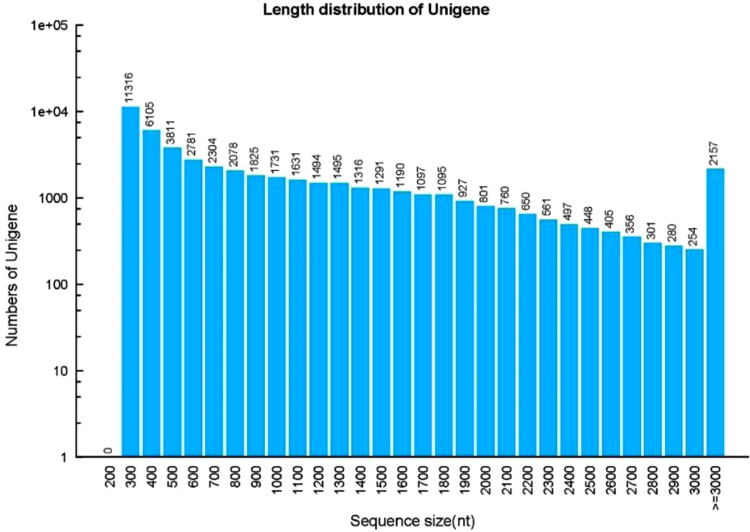
Length distributions of the de novo assembly for unigenes. The length distribution of unigenes were counted with an interval of every 100 bp from 300 bp to 3000 bp. *X*-axis indicates sequence size (nt), *Y*-axis indicates number of assembled contigs and unigenes.

**Fig. 2:**
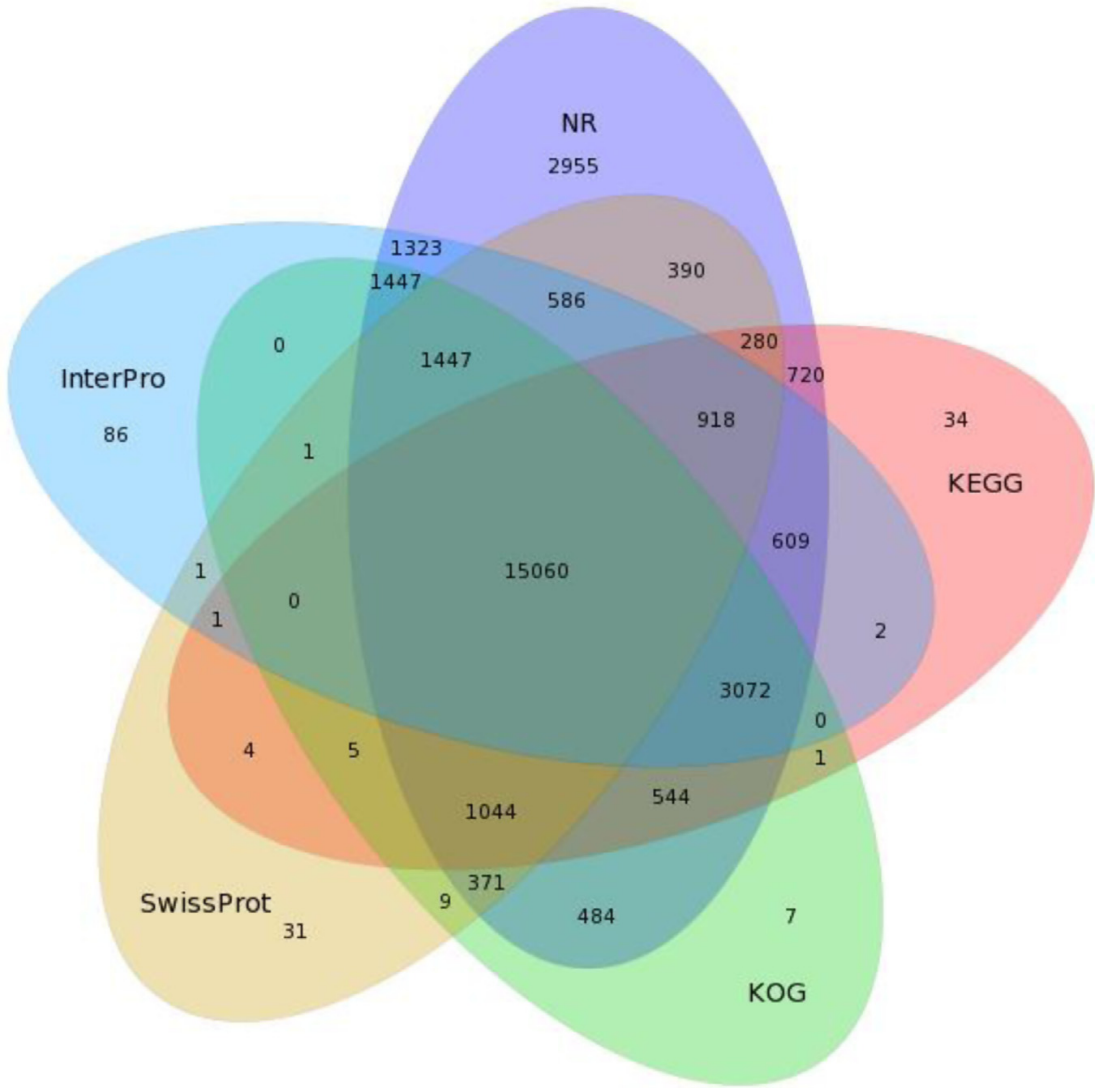
Venn diagram shows commonality and difference of annotation based on NR, KEGG, Swiss-Prot, InterPro and KOG.

**Fig. 3:**
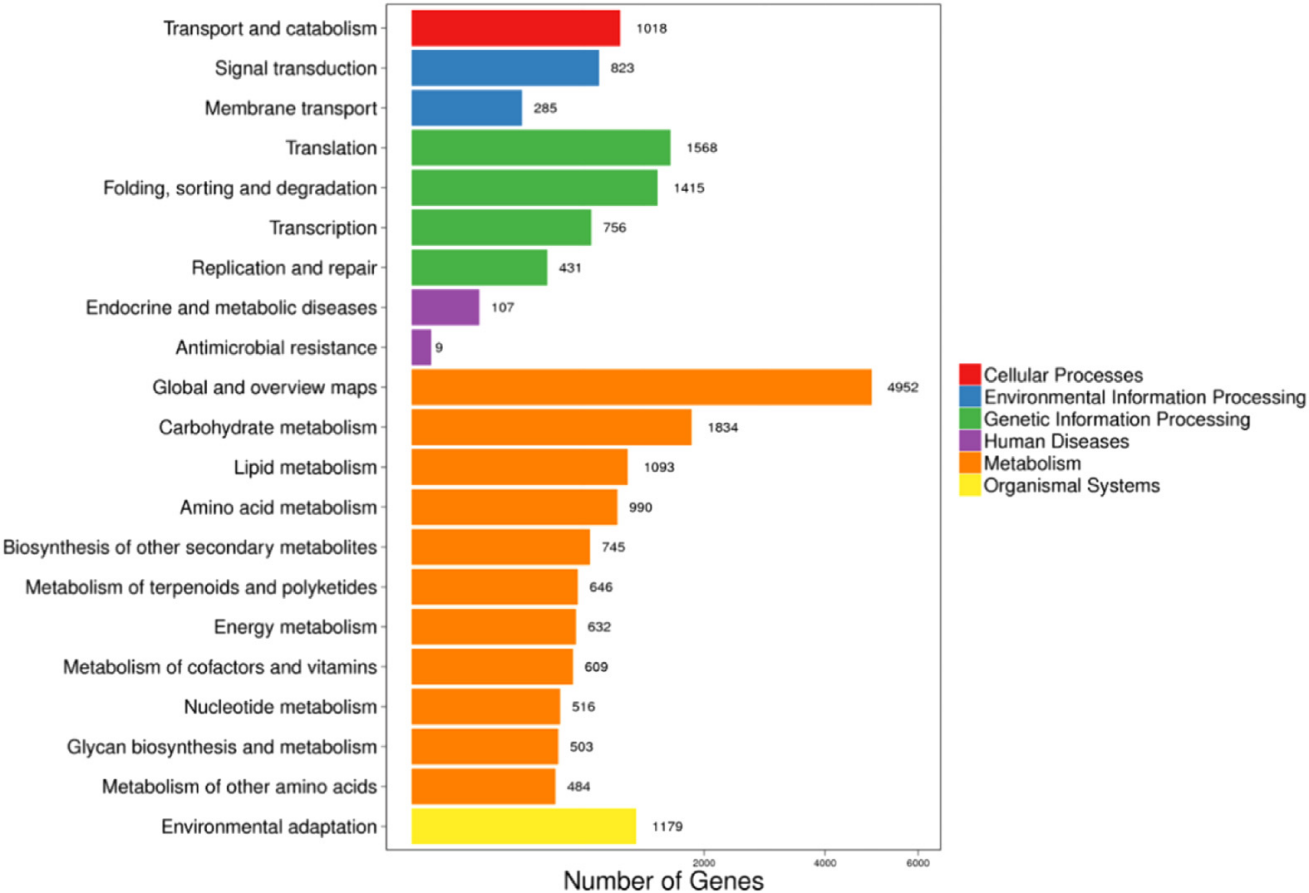
KEGG annotation of unigenes.

Here we report a de novo transcriptome assembly of *A. venetum*. Our aim was to obtain a high quality reference transcriptome of *A. venetum* leaves, elucidate the molecular pathway of fiber and flavonoids synthetize, stress resistance, and find candidate genes of these process (see Table 1, Table 2, Table 3 and Fig. 1, Fig. 2, Fig. 3).

**Table 1:** Summary of the transcriptome sequencing and assembly.

Assembly statistic	*Apocynum venetum*
Total Clean Bases(Gb)	6.57
Clean Read Q20 (%)	95.97
Number of assembled reads	63,906
Total Length of assembled reads	58,605,303
Number of unigenes	50,957
Total Length of unigene (bp)	51,426,191
Average unigene length (bp)	1009
GC (%)	40.42
Unigene N50 (bp)	1632

**Table 2:** Unigenes functional annotation by various databases.

Index	*Apocynum venetum*
Unigenes annotated in Nr	31,250
Unigenes annotated in Nt	21,507
Unigenes annotated in Swissport	20,148
Unigenes annotated in KEEG	22,294
Unigenes annotated in KOG	23,492
Unigenes annotated in Interpro	24,553
Unigenes annotated in GO	10,483

**Table 3:** General statistics of SSR identified transcriptome.

Item	Marker
Total number of identified SSR	7579
Number of SSR containing sequences	6340
Number of sequences containing> 1 SSR	1040
Mononucleotide	3003
Dinucleotide	2730
Trinucleotide	1512
Tetranucleotide	43
Pentanucleotide	112
Hexanucleotide	179

The de novo transcriptome assembly of *A. venetum L.,* and the SRA records is accessible with the following link: https://www.ncbi.nlm.nih.gov/sra/SRP151546.

## 2 Experimental design, materials and methods

### 2.1 Plant materials

The seeds of *A. venetum* were collected from Xinjiang Province, China, in November 2016. Seeds were surface-sterilized by rinsing in 70% (v/v) ethanol for 60 s, then in 5% (v/v) sodium hypochlorite (NaClO) for 30 min while rocking on a platform, and washed in distilled water for 8 min. The seeds were allowed to germinate and grow for 30 days in half-strength MS agar medium inside a growth chamber with a 14 h light/10 h dark cycle, air temperature of 25 °C, photon flux density (PFD) of 280 mol m^−2^ s^−1^. The leaves of *A. venetum* were collected, immediately frozen in liquid nitrogen, and stored at -80 °C until use. Total RNA was extracted using TRIzol Reagent (Invitrogen, LifeTechnologies, USA) following the manufacturer׳s instructions, then rtreated with DNase I (Invitrogen, Life Technologies, USA). The RNA integrity was verified using an Agilent 2100 BioAnalyzer (Agilent, USA).

### 2.2 RNA sequencing

RNA-Seq libraries were constructed using the RNA Library Prep Kit for Illumina using to the manufacturer׳s instructions (NEB, USA). Library quality was assessed on the Agilent Bioanalyzer 2100 system. The libraries were sequenced on the BGIEQ-500 platform (BGI, CHN) based on sequencing by synthesis with 100 bp paired-end reads (BGI Technologies, Shenzhen). All RNA-Seq data were deposited in National Center for Biotechnology Information (NCBI) with the accession number SRP151546.

### 2.3 Leaf transcriptome assembly and gene functional annotation

The raw reads were firstly filtered and combined to form longer fragments, then de novo assembled into unigenes using the short read assembly program Trinity with default settings [4], [5]. Functional annotation of the unigenes was performed by searching the following databases: Nr; Pfam; KOG/COG; Swiss Prot; KEGG; and GO. The information on the annotation was summarized and the distribution of unigenes was illustrated by Venn diagram (Fig. 2).

### 2.4 Identification of SSR markers

Using the MISA software [6], 6,340 unigenes containing 7,579 SSRs were identified, of which 1040 sequences contained more than one SSR.
