# Supplementary material for: Data set for transcriptome analysis of Apocynum venetum L
Source: Data Brief. 2018 Sep 5;20:1739–44. doi: 10.1016/j.dib.2018.08.207 (PMC6160392; doi:10.1016/j.dib.2018.08.207)
Supplement: Supplementary file 1 — Supplementary material [file mmc1.docx]

**Conflict of interest**

Authors declare no conflict of interest.
